# The interplay between oxidative stress and autophagy in chronic obstructive pulmonary disease

**DOI:** 10.3389/fphys.2022.1004275

**Published:** 2022-09-26

**Authors:** Xiaoyu Zhao, Qiang Zhang, Rui Zheng

**Affiliations:** Department of Pulmonary and Critical Care Medicine, Shengjing Hospital of China Medical University, Shenyang, China

**Keywords:** chronic obstructive pulmonary disease, autophagy, oxidative stress, interplay, molecular mechanism

## Abstract

Autophagy is a highly conserved process that is indispensable for cell survival, embryonic development, and tissue homeostasis. Activation of autophagy protects cells against oxidative stress and is a major adaptive response to injury. When autophagy is dysregulated by factors such as smoking, environmental insults and aging, it can lead to enhanced formation of aggressors and production of reactive oxygen species (ROS), resulting in oxidative stress and oxidative damage to cells. ROS activates autophagy, which in turn promotes cell adaptation and reduces oxidative damage by degrading and circulating damaged macromolecules and dysfunctional cell organelles. The cellular response triggered by oxidative stress includes changes in signaling pathways that ultimately regulate autophagy. Chronic obstructive pulmonary disease (COPD) is the most common lung disease among the elderly worldwide, with a high mortality rate. As an induced response to oxidative stress, autophagy plays an important role in the pathogenesis of COPD. This review discusses the regulation of oxidative stress and autophagy in COPD, and aims to provide new avenues for future research on target-specific treatments for COPD.

## Introduction

**FIGURE 1:**
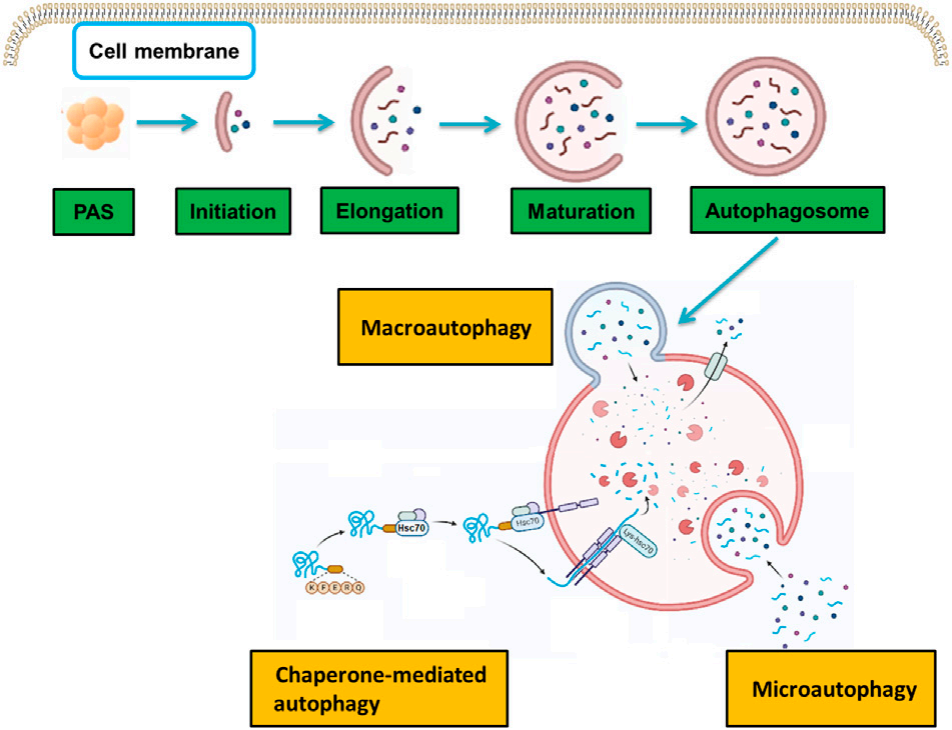
Molecular mechanisms in the three types of autophagy. There are three types of autophagy: macroautophagy, microautophagy, and chaperone-mediated autophagy. Each type involves a specific molecular mechanism of delivery to lysosomes.

Autophagy is an evolutionary cellular process that maintains cellular homeostasis through lysosome-mediated degradation of intracellular lipids, proteins, and organelles. There are three main types of autophagy, i.e., macroautophagy, microautophagy, and chaperone-mediated autophagy, each of which involves a specific mechanism of delivery to lysosomes (Figure 1). Macroautophagy, which is what “autophagy” usually refers to, is the most typical form of autophagy and begins at the initial stage of the development of autophagosomes. Microautophagy is distinct from macroautophagy that happens via lysosomal membrane invaginations, creating tubes and directly engulfing cytoplasm. Chaperone-mediated autophagy is a selective form of autophagy, in which the soluble proteins are selectively sequestered and delivered to lysosomes.

Chronic obstructive pulmonary disease (COPD) is a preventable and treatable disease characterized by persistent airflow limitation. Most of this airflow limitation develops progressively and is associated with an abnormal inflammatory response to harmful gases, such as cigarette smoke (CS) or harmful particulate matter in airway and lung tissues. However, its etiology remains poorly understood and might be the result of long-term interactions between various environmental factors (*e.g.*, smoking, exposure to occupational dust and chemicals, air pollution, and infection) and inherent factors in the body (*e.g.*, immune dysfunction, airway hyper-responsiveness, and aging).

Notably, COPD is a common, recurrent respiratory disease, especially among the elderly (Yao et al., 2021) and a major cause of death worldwide (Aggarwal et al., 2016). It is characterized by chronic bronchitis, chronic airway obstruction, airway remodeling and emphysema, all of which lead to a progressive decline in pulmonary function (Wang et al., 2018). Chronic inflammation leads to airway stenosis and destruction of the lung parenchyma, resulting in loss of elastic recoil and mucociliary function. Patients with COPD primarily present with respiratory symptoms, such as chronic cough, sputum production, shortness of breath or dyspnea, wheezing, and chest distress (Tan et al., 2019).

Smoking remains the principal risk factor for COPD. Numerous studies have shown that cigarette smoke exposure (CSE) is the primary cause of the onset and progression of COPD, leading to excessive inflammation of the airways, alveoli, and capillaries (Vogelmeier et al., 2017). CS is composed of particles in the solid (tar) and gas phases (free radicals, toxic gases, and volatile organic compounds), which generate reactive oxygen species (ROS), such as superoxide anion (^.^O_2_^−^) and hydroxyl radical (^.^OH). These cause damage to biological systems (Valavanidis et al., 2009) and many studies have shown that patients with COPD exhibit high levels of inflammation and oxidative stress in their lungs (Zuo et al., 2014). Oxides primarily include superoxide anion, hydroxyl radical, hypochlorous acid, hydrogen peroxide (H_2_O_2_), and nitric oxide, which can act directly on and destroy many biomacromolecules, such as proteins, lipids, and nucleic acids, leading to cell dysfunction or death. These oxides can also destroy the extracellular matrix, cause protease-antiprotease imbalance, promote inflammatory responses, such as activation of the NF-κB transcription factor, participate in the transcription of various inflammatory mediators, such as interleukin-8 (IL-8) and tumor necrosis factor-α (TNF-α), and induce the expression of nitric oxide synthase (NOS) and epoxide hydrolase.

An increasing body of evidence has shown that autophagy is dysregulated in COPD. Deficits in autophagy have been reported to lead to failure in the clearance of proteins and organelles damaged by oxidative stress, thereby accelerating the development of COPD (Vij et al., 2018). In contrast, overactive autophagy has been shown to lead to increased cell death or apoptosis and loss of cilia, ultimately leading to emphysema (Liu and Levine, 2015). Dysregulation of autophagy due to smoking, environmental insults, aging, or other factors can lead to the formation of aggressors and more ROS, which contribute to the pathogenesis of COPD (Tan et al., 2019). In this review we aimed to elucidate the relationship between autophagy and COPD under oxidative stress, focusing on how autophagy develops and through which mechanisms. The molecular mechanism of autophagy, the relationship between autophagy and oxidative stress, and the mechanism of autophagy dysregulation under oxidative stress in COPD were investigated.

## The concept of autophagy

In 1955, the Belgian cytologist and biochemist Christian de Duve was the first to report the discovery of a new type of organelle isolated from a rat liver homogenate. This new type of organelle was found to contain acid phosphatases and hydrolases that were most active under low pH conditions (De Duve et al., 1955) and it was termed as lysosome. In 1956, Novikoff *et al.* observed the subcellular localization of lysosomes in rat liver using electron microscopy and found that the lysosome fraction contained dense corpuscles with lumens and outer membrane structures, as well as many dense particles that had not been previously described (Novikoff et al., 1956). In 1957, Clark *et al.* observed the kidney tissue of newborn mice with Swiss albino mice by electron microscopy, and discovered the presence of many large vacuoles in cells, in addition to lysozymes, which contained amorphous substances and mitochondria-sized dense inclusions. These large vacuoles, which were identified as double-membrane structures lacking hydrolase, were later named autophagosomes (Clark, 1957; Deter and De Duve, 1967). At a 1963 international conference on lysosomes, de Duve proposed the name “autophagy,” originating from Greek, meaning “self-eating”, for the process of degrading cytoplasmic material through the fusion of lysosomes with autophagosomes (Ueno and Komatsu, 2017).

Unfortunately, research on autophagy progressed slowly in the following 30 years and did not attract widespread attention due to technical limitations. A turning point occurred in the 1990s, when the laboratory of Yoshinori Ohsumi discovered the process of autophagy in yeast. The group developed a new method for monitoring autophagy by constructing cells in which vacuolar phosphatase was expressed in its inactive precursor form. Phosphatase was only active after its processing in the vacuole, allowing the measurement of the activity of mature phosphatase, and thus its quantitatively correlation with the level of autophagy (Noda et al., 1995). This method prompted the study of various genes involved in the regulation of autophagy. The group began genetic screening of autophagy-deficient mutants (Tsukada and Ohsumi, 1993). and identified multiple autophagy-related genes in a yeast model. In order to avoid confusion, both this and other research groups that identified autophagy-deficient mutants, developed a uniform gene nomenclature system, naming these genes autophagy-related genes (ATG), and studied the interaction between the proteins they encode and their roles in autophagy (Thumm et al., 1994; Harding et al., 1995). Accordingly, the identification of ATG genes in yeast led to the rapid development of research on autophagy. Nearly all functional homologues of yeast autophagy-related genes have been found in higher eukaryotes and humans, further confirming their primary role in the degradation pathways for organelles and proteins (Sheng and Qin, 2019).

The process of autophagy refers primarily to the formation and degradation of membranes. Appearance of autophagosomal membranes is associated with the general classification of autophagy into three categories, depending on the pathway through which the cellular material is delivered to lysosomes. For instance, in macroautophagy, the material is encapsulated in endoplasmic reticulum-derived membranes that subsequently fuse with lysosomes for degradation (Stavoe and Holzbaur, 2020). In this review, autophagy refers primarily to macroautophagy, which is characterized by the formation of autophagosomes (Button et al., 2017), whereas, in microautophagy, long-lived proteins in the cell are directly engulfed by lysosomes and degraded (Ghosh et al., 2016). Finally, in chaperone-mediated autophagy (CMA) (Oshima et al., 2019), soluble intracellular proteins are bound by molecular chaperones and delivered to lysosomes, where they are enzymatically digested. Autophagy is also divided into nonselective and selective autophagy according to the nutritional status. The former occurs under starvation conditions, whereas the latter occurs under nutrient-rich conditions (Takáts et al., 2019; Martens and Behrends, 2020). Common types of selective autophagy include mitochondrial autophagy (mitophagy) and peroxisome autophagy (Eun et al., 2018; Hasnat et al., 2019; Cao et al., 2021). The macroautophagy and microautophagy belong to the nonselective autophagy, while CMA is a selective autophagy.

Autophagy is a process of stress adaptation to the influence of external factors mediated by autophagy-related genes (ATGs) (Kang et al., 2018). In particular, rough endoplasmic reticulum lacking ribosomes partially detaches from the double membrane and gradually encloses some cytoplasm, organelles, proteins, and other necessary components for degradation and then fuses with lysosomes to form autophagosomes, the content of which is degraded by lysosomal enzymes. The primary function of autophagy is to recycle amino acids and monosaccharides through the degradation of endogenous biological macromolecules. Consequently, autophagy is a form of catabolism, which is a highly conserved energy-dependent process of pathophysiological adaptation (Galati et al., 2019). In addition, a large body of evidence has suggested that autophagy is activated under stress conditions, such as starvation or energy failure, highlighting its particular importance in maintaining cell homeostasis and survival in the absence of nutrients (Sheng and Qin, 2019; Cao et al., 2021).

Autophagy has attracted increasing attention and has become a popular topic of biological, medical, botanical, and microbiological research in recent years. Many researchers have investigated the relationship between non-selective and selective autophagy and various pathophysiological states in humans, as well as the molecular mechanisms of the regulation of autophagy in cancer, neurodegenerative diseases, cardiovascular diseases, immune reactions, development, and aging. This review focuses on the regulation of autophagy in COPD under oxidative stress.

## Molecular mechanisms of autophagy

**FIGURE 2:**
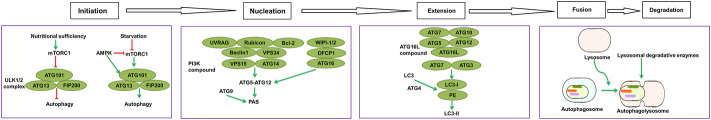
List of molecular entities involved in autophagy. ATGs-regulated autophagy system is divided into five stages: initiation, nucleation, expansion, fusion, and degradation.

In mammals, autophagy regulates the response to environmental signals through a protein network known as the core autophagy machinery. The regulatory system is composed of the products of over 30 autophagy-related genes (ATGs), which are homologues of similar proteins (Atgs) originally identified in yeast (He and Klionsky, 2009). In particular, ATGs are involved in every step of autophagy, which is divided into five stages: initiation, nucleation, expansion, fusion, and degradation (Figure 2).

Induction of autophagy is triggered by a variety of extracellular and intracellular stimuli, such as rapamycin and other mTOR inhibitors (Mizushima, 2007). Hypoxia (Mazure and Pouyssegur, 2010), oxidative stress (Filomeni et al., 2015), and nutritional starvation, including total consumption of amino acids (Levine and Klionsky, 2004). The initiation stage includes the activation and assembly of signaling components that trigger processes in response to environmental cues, including nutrient and energy levels and the accumulation of damaged substrates (Klionsky, 2007; Eskelinen and Saftig, 2009; Rabinowitz and White, 2010; Ravikumar et al., 2010). The autophagy pathway is downregulated by nutritional and growth factor-related signals, and is upregulated by starvation or energy consumption in the target of rapamycin (mTOR). mTOR pathway is located in a macromolecular complex (mTOR complex 1 [mTORC1]) (Nakahira et al., 2014). Among the known downstream regulatory targets of mTORC1, mammalian uncoordinated 51 (Unc-51)-like protein kinases, such as the autophagy activating kinase 1 or 2 (ULK1/2) complex, is the primary regulator of autophagy initiation (Chan et al., 2007). In turn, mTORC1 negatively regulates the mammalian Unc-51-like protein kinase (ULK1/2) complex, which in humans includes ATG13, ATG101, and focal adhesion kinase family interacting protein of 200kD (FIP200) (Corona Velazquez and Jackson, 2018). ULK1 is a member of the ULK1-4 kinase family and the most important component in autophagy (Tan et al., 2019). Under adequate nutritional conditions, the interaction between mTORC1 and ULK1 inhibits the activity of ULK1, thereby inhibiting the activation of autophagy (Hosokawa et al., 2009). When mTOR is inhibited by starvation or rapamycin, ULK1 and ULK2 are activated and ATG13 and FIP200 are phosphorylated; this is the key step in starvation-induced autophagy in mammalian cells (Chang and Neufeld, 2009; Hosokawa et al., 2009; Mizushima, 2010; Hurley and Young, 2017). Autophagy is positively regulated through energy consumption by 5′-adenosine monophosphate (AMP)-activated protein kinase (AMPK). This kinase regulates mTORC1 (Jung et al., 2010; Kim et al., 2011) by inactivating it, while also phosphorylates and actives ULK1 to promote autophagy, leading to the degradation of cellular components for the production of ATP (Egan et al., 2011; Kim et al., 2011; Loffler et al., 2011). Formation of the ULK1/2 complex triggers the initiation of the formation of double-membrane autophagosomes (Ornatowski et al., 2020).

In the nucleation stage, the autophagy precursor structure develops from subcellular membranes that form into a phagosome in a separate membrane. The origin of this structure is still not fully understood, but it is thought to be derived from the endoplasmic reticulum or other intramembrane compartments (Brest et al., 2010). The nucleation step of the formation of autophagosomes requires an additional regulatory complex, the class III phosphatidylinositol 3-kinase (PI3K) complex, which is composed of Beclin 1, phosphatidylinositol 3-kinase catalytic subunit type 3 (VPS34), phosphoinositide-3-kinase regulatory subunit 4 (VPS15), and ATG14 (Mack et al., 2012). In mammalian cells, Beclin 1 is the central component of the PI3K complex that interacts with various proteins, such as ATG14, UV radiation resistance associated (UVRAG), Rubicon, and Bcl-2 to regulate the size and number of autophagosomes (Itakura et al., 2008; Li et al., 2012; Fogel et al., 2013), whereas, BCL-2 family proteins interact with Beclin 1 to inhibit autophagy (Pattingre et al., 2005; He and Levine, 2010). Activation of Beclin 1 is responsible for the production of phosphatidylinositol 3-phosphate (PI3P), which is necessary for the formation of autophagosomes (Burman and Ktistakis, 2010). Activation of Beclin 1 and its associated proteins enhances the activity of PI3P (Racanelli et al., 2020), which regulates the formation of autophagosomes by recruiting protein factors, including WD repeat domain phosphoinositol-interacting protein (WIPI-1/2; Atg18 in yeast) and double FYVE-containing protein 1 (DFCP1) (Proikas-Cezanne et al., 2004; Polson et al., 2010; Ravikumar et al., 2010), as well as ATG16L (Ornatowski et al., 2020). Consecutively, the ATG5-ATG12 system is coupled to the phagosome assembly site (PAS) (Proikas-Cezanne et al., 2015), while Atg9-containing vesicles produced through the secretory pathway are recruited to the PAS, allowing the delivery of additional lipids and proteins and contributing to membrane expansion (Noda, 2017). Activated ULK1 also enhances the localization of Atg9 by phosphorylation its site on ATG9 at Ser14 (Zhou et al., 2017), which is considered to be a membrane carrier protein for autophagosome formation to PAS (Mack et al., 2012).

In the expansion stage, the separate membrane expands to surround and engulf the substances to be degraded, forming complete autophagic vacuoles or autophagosomes with a double-membrane structure (Kelekar 2005; Ravikumar et al., 2010). After the formation of autophagosomes, their expansion requires 2 ubiquitin-like coupling systems, namely the ATG5-ATG12 and microtubule-associated protein 1A/1b light chain 3 (LC3)/Atg8 coupling system (Nakatogawa 2013). In particular, ATG7 and ATG10 couple ATG5 to ATG12, forming a large multimeric ATG16L complex through noncovalent binding with ATG16L that participates in the expansion of the autophagic membrane (Yang and Klionsky, 2010). These factors are subsequently separated from autophagosomes during maturation. Concomitantly, ATG4 cleaves LC3 at its C-terminal into its LC3-I form, which has an exposed lipid coupling site at a glycine residue in its C-terminal. This facilitates the ATG7-and ATG3-mediated coupling of a phosphatidylethanolamine (PE) molecule to the C-terminal glycine residue of LC3-I for the production of LC3-II (Tan et al., 2019). In mammals, the conversion of LC3-I (uncoupled cytoplasmic form) to LC3-II (coupled autophagosome membrane-associated phosphatidylethanolamine form) is considered a hallmark of the formation of autophagosomes (Mizushima et al., 2010; Ravikumar et al., 2010). Subsequently, LC3-II attaches to both sides of the phagosome membrane and is removed from the outer membrane before the autophagosome fuses with a lysosome (Szumiel, 2011). Moreover, the recruitment of LC3-II to autophagosomes is mediated by the ATG5-ATG12-ATG16L complex, which also contributes to the coupling with LC3 (Hanada et al., 2007).

Finally, after initiation, nucleation, and expansion are completed, the expanded membrane closes around the cargo to form a complete autophagosome, with the following fusion of the autophagosome with the lysosome, resulting in the formation of an autophagolysosome with degradative ability. Of note, mature autophagosomes can also fuse with endocytic vacuoles (endosomes) to form phagosomes, which also develop into lysosomes (Ryter and Choi 2010). The PI3K complex can activate the GTPase Rab7, thereby promoting fusion with the lysosome (Liang et al., 2008). In addition, the lysosome-associated membrane protein LAMP-2A is necessary for the fusion of the autophagosome with a lysosome (Nakahira et al., 2014).

The following step is degradation, where a series of lysosomal degradative enzymes, such as cathepsin and other acid hydrolases, digest the encapsulated contents of the autophagolysosome. The digested content is then released into the cytoplasm as a new energy source for cell survival or is reused in biosynthetic pathways (Kelekar 2005; Klionsky, 2007; Eskelinen and Saftig, 2009; Rabinowitz and White, 2010; Ravikumar et al., 2010). In addition, autophagy helps to remove damaged organelles, such as mitochondria, thereby participating in other major mechanisms for cell survival (Williams and Ding, 2018).

## Relationship between autophagy and oxidative stress

Oxidative stress refers to a state in which the oxidative and antioxidant effects in the body are imbalanced toward oxidation, leading to the inflammatory infiltration of neutrophils, increased secretion of proteases, and production of large amounts of oxidation intermediates. Oxidative stress is produced by free radicals in the body and is considered to be a major factor involved in aging and disease. Oxygen is converted into reactive oxygen (ROS) and reactive nitrogen (RNS) species through enzymatic and nonenzymatic processes, leading to damage to proteins, lipids, and DNA (Rogers and Cismowski 2018). Oxidative stress reflects an imbalance in ROS due to increased production of ROS or decreased local antioxidant defense or both. Therefore, a balance between the production and clearance of ROS is necessary to avoid cell damage and safeguard human health (Pizzino et al., 2017). ROS is produced in cells during aerobic metabolism under physiological or pathological states or through redox processes during exposure to environmental triggers or drugs, and usually includes oxygen free radicals such as superoxide anion (^.^O_2_^−^), hydroxyl radical (^.^OH), and hydrogen peroxide (H_2_O_2_) (Ornatowski et al., 2020), while RNS include NO, nitrogen dioxide (^.^NO_₂_), and peroxynitrite (^.^ONOO^−^). As mitochondria are both the principal source of ROS and the functional target of the production of ROS, the levels of ROS produced by mitochondria under physiological conditions are low. However, the production of ROS is significantly increased under pathological conditions and during aging (Nakahira et al., 2014; Ornatowski et al., 2020). There are 2 types of antioxidant systems in the body, the enzymatic antioxidant system, which includes superoxide dismutase (SOD), catalase (CAT), and glutathione peroxidase (GSH-Px), and the nonenzymatic antioxidant system, which includes vitamin C, vitamin E, and glutathione. These antioxidant systems are known to antagonize and detoxify ROS to maintain redox homeostasis in the cell (Venditti et al., 2013; Netto and Antunes, 2016). Studies have shown that oxidative stress or increased generation of ROS promotes the activation of autophagy (Kiffin et al., 2006, Chen et al., 2007a, Chen et al., 2007b). As such, autophagy might be considered a secondary defense against oxidative stress because it promotes the turnover of damaged or modified substrates, such as proteins. As approximately 90% of ROS is derived from the respiratory chain in the inner mitochondrial membrane (Murphy, 2009; Shadel and Horvath, 2015), autophagy potentially plays a central role in the response to oxidative stress in mitochondrial quality control. In addition, studies have shown that autophagy might be cross-regulated with the antioxidant response in mammals, and the transcription of certain autophagy-related proteins might be directly regulated by the redox state (*e.g.*, ATG4B) (Scherz-Shouval et al., 2007) or oxidative stress (*e.g.*, p62) (Jain et al., 2010). Interestingly, the p62 ubiquitin-binding protein has been shown to enhance the dissociation of Keap-1-Nrf2 and promote the degradation of Keap1 through p62-dependent autophagy, further stimulating the antioxidant response (Dodson et al., 2015; Kageyama et al., 2018). In addition, sestrins (SESNs) which are proteins involved in protecting cells from oxidative stress, have also been found to promote the p62-dependent autophagic degradation of Keap-1 (Chen et al., 2019). Endogenously produced ROS is also considered a signaling mediator involved in autophagy induced by a variety of stimuli, including nutrient consumption and pro-inflammatory mediators. In summary, there is a close relationship between oxidative stress and autophagy, with oxidative stress inducing autophagy through a variety of mechanisms, and vice versa.

## Autophagy and antioxidant responses

Studies have shown that cellular antioxidant responses can cross-regulate autophagy. The nuclear factor erythroid 2-related factor 2 (Nrf2) oxidative stress response transcription factor is the primary regulator of cellular antioxidant defense (Nakahira et al., 2014). The function of Nrf2 in controlling the transcription of antioxidant genes is known to be mediated by its interaction with antioxidant response (ARE) (Kaspar et al., 2009) or electrophilic response (EpRE) elements (Mitsuishi et al., 2012). Under normal growth conditions, Nrf2 dissociates from Kelch-like ECH-related protein-1 (Keap-1), its cytoplasmic inhibitor, which acts as an adaptor protein for the CUL3-ubiquitin E3 ligase complex that ubiquitinates Nrf2 (Suzuki and Yamamoto, 2017). Under oxidative stress, Nrf2 dissociates from Keap1, and translocates to the nucleus, where it binds to AREs in the promoter region of antioxidant genes (Kobayashi et al., 2006; Jain et al., 2010; Suzuki and Yamamoto, 2017). The p62 autophagy cargo adaptor protein is a newly discovered Nrf2 target gene activated through many stress response pathways, including nutrient deprivation, mitochondrial damage, and oxidative stress (Jain et al., 2010). In this process, p62 interacts with the Nrf2 binding site on Keap1 to promote the release of Nrf2 from Keap-1, which subsequently activates the transcription of Nrf2 target genes (Ichimura et al., 2013). Some studies have shown that the Keap1-p62 complex formed in this process is recruited into autophagosomes by LC3 and then degraded via autophagy (Jain et al., 2010; Taguchi et al., 2012; Ichimura et al., 2013). Under conditions of oxidative stress, Nrf2 induces the expression of the p62 gene, leading to a further increase in Nrf2 (Jain et al., 2010; Komatsu et al., 2010). Therefore, p62 participates in a positive feedback loop by increasing autophagy and maintaining the antioxidant effect of Nrf2 (Jain et al., 2010). SESNs are proteins involved in protecting cells from oxidative stress by promoting the antioxidant adaptive response in cells through the activation of transcription factors, such as p53, Nrf2, AP-1, and FoxOs (Ornatowski et al., 2020). An increasing body of evidence has shown that SESNs are positive regulators of autophagy under different environmental stresses (Maiuri et al., 2009; Bae et al., 2013; Hou et al., 2015). In particular, overexpression of SESN1 or SESN2 has been reported to induce the autophagic degradation of Keap1 and increase the activity of Nrf2 (Bae et al., 2013). Co-immunoprecipitation analysis showed that SESN2 directly interacts with p62, promoting the autophagic degradation of the p62-dependent target Keap1, and preventing oxidative damage (Bae et al., 2013).

## Mutual regulation of autophagy and oxidative stress

Under oxidative stress, autophagy is activated to protect cells from apoptosis (Mizushima, 2009), whereas, inhibition of autophagy leads to accumulation of oxidative stress damage and cell death (Lee et al., 2012). Autophagy eliminates the source of oxidative stress by removing damaged cellular components (Kiffin et al., 2006; Filomeni et al., 2015), thereby protecting cells from oxidative damage. Moreover, it plays an essential role in cellular antioxidant defense by maintaining mitochondrial quality control and mitochondrial phagocytosis, preventing the production of pathological mtROS, and clearing damaged mitochondria. Increased production of ROS activates the HIF-1α transcription factors p53, FOXO3, and Nrf2, which in turn induce the transcription of BNIP3, NIX, TIGAR, LC3, and p62 (Lee et al., 2012). ROS has been demonstrated to regulate autophagy through mTOR-dependent pathways in the cytoplasm (Scherz-Shouval and Elazar, 2011; Zhang et al., 2014).

Interestingly, ROS activates autophagy by either inhibiting the PI3K-Akt-mTOR pathway or activating AMPK to inhibit the mTOR signaling pathway (Zhang et al., 2013). Mitochondrial phagocytosis selectively degrades mitochondria through the PTEN-induced protein kinase (PINK) 1-Parkin and BNIP3-NIX-FUNDC1 pathways. PINK1 and Parkin appear to be the first targets in the signaling pathway, activating the mitochondrial quality control pathway in response to mitochondrial damage (Harper et al., 2018). Hypoxia, stress, and other stimuli trigger mitochondrial phagocytosis. More specifically, mitochondrial phagocytosis is activated by hypoxia through the induction of the BNIP3, NIX, and FUNDC1 adaptor proteins, which are localized to the outer mitochondrial membrane and contain a LC3 interaction motif to promote the recruitment of autophagy-associated mechanisms (Bellot et al., 2009). In conclusion, oxidative stress and autophagy regulate each other, with autophagy regulating redox homeostasis through the Nrf2 and SESNs antioxidant pathways.

## Regulation of autophagy during oxidative stress in COPD

### COPD and oxidative stress

COPD is a respiratory disease caused by direct and long-term exposure to toxic particulates or gases. It triggers airway or alveolar abnormalities, leading to symptoms of chronic bronchitis and emphysema, which usually manifest as persistent respiratory symptoms and airflow restriction (Rodriguez-Roisin et al., 2017). CS is a major risk factor for COPD, and thus the mortality rate of COPD among smokers is higher than that of non-smokers (Vestbo et al., 2013). Notably, CS is a complex mixture containing 4,700 chemical components, including carbon monoxide, heavy metals, aldehydes, aromatic hydrocarbons, free radicals, and other oxidizing compounds (Church and Pryor, 1985). Studies have shown that although e-cigarettes produce fewer toxic substances than traditional cigarettes, they also contain nicotine, also making them potentially harmful to the lungs (Zhang et al., 2020a). Our previous studies found that exposure to CS significantly reduced the expression levels of protein tyrosine phosphatase-like Adomain containing 2 (PTPLAD2) and ubiquitin specific peptidase 49 (USP49) in BEAS-2B cells, suggesting that these genes might play a key role in CSE-induced COPD (Zhang et al., 2020b). The primary targets of inhaled CS are airway and alveolar epithelial cells (Racanelli et al., 2018). Exposure to CS, uncontrolled chronic inflammation and oxidative stress are the main drivers of the pathogenesis of COPD in airway epithelial cells, and are involved in many forms of regulated cell death (*i.e.*, apoptosis, necroptosis, and autophagy) (Racanelli et al., 2020). Smoking is a major cause of systemic oxidative stress, excessive inflammation, and emphysema. Patients with COPD are continuously exposed to high levels of oxidative stress and lung inflammation, which can lead to airway obstruction and destruction of the lung parenchyma (Tan et al., 2019). The large oxidative burden, caused by mitochondrial dysfunction, has been confirmed to be the principal cause of abnormal response and refractory inflammation due to exposure to CS (Jiang et al., 2017). In addition, the free radical theory states that oxidative stress is the primary driving factor leading to cellular aging (Radak et al., 2005). Autophagy is a process of homeostatic degradation of organelles or proteins involved in oxidative stress damage, and also plays a role in regulating inflammation by regulating the development and survival of inflammatory cells (Qian et al., 2017).

A number of studies have confirmed the importance of oxidative stress for inducing lung disorders. These studies have determined the existence of free radical biomarkers that induce lung inflammation and autoimmune responses and damage in patients with COPD. Thus, correcting these oxidant/antioxidant imbalances can prevent the progression of COPD (Yao and Rahman, 2011). The principal sources of ROS and RNS in the lungs are the environment and cells (Yao and Rahman, 2011). Injury and exposure to triggers stimulates the production of endogenous oxidation products by epithelial cells, endothelial cells, airway cells, and alveolar macrophages, and also recruits inflammatory cells to the lungs, generating additional oxidative stress (Rogers and Cismowski, 2018). ROS and RNS are also known to be produced by various inflammatory and structural cells in the airway. One of the characteristics of COPD is its inflammatory immune response, which is characterized by the recruitment and activation of epithelial cells and macrophages, neutrophils, monocytes, and lymphocytes. In particular, once inflammatory cells are recruited into the airway, they are activated, producing ROS (Yao and Rahman, 2011). Some researchers have found that CS participates in the progression of COPD by inducing the M1 and M2 polarization of macrophages (Feng et al., 2020). Rosiglitazone was reported to inhibit the CS-induced M1 polarization of macrophages and decrease the M1/M2 ratio, thereby improving emphysema and inflammatory reactions due to exposure to CS (Feng et al., 2021). Oxidases, including nicotinamide adenine dinucleotide phosphate (NADPH) oxidase (NOXs), are the primary source of oxidative stress in the lungs. Studies have found that NOXs produce a large amount of oxidative agents that protect the lungs (Al Ghouleh et al., 2011; Lee et al., 2012). Lung hypoxia, ischemic injury, and airway inflammation irreversibly convert peroxisomal xanthine dehydrogenase into xanthine oxidase, which is the principal source of the production of superoxide and is also involved in COPD process (Rogers and Cismowski 2018). In addition, the pulmonary endothelium also upregulates the production of NO by increasing the NOS activity. Besides, ·NO is a highly reactive species that reacts rapidly with O_2_^−^, producing the more destructive peroxynitrite (ONOO^−^) and other RNS (Rogers and Cismowski 2018). The balance of autophagy plays an important role in maintaining the dynamics of the intracellular environment. Nonetheless, COPD can cause cellular damage severe enough to trigger autophagy in lung cells (Dan Dunn et al., 2015; Aggarwal et al., 2016; Chen et al., 2016). As CS inhalation inactivates proteases necessary for protecting the lungs, the development and progression of COPD have been closely related to the oxidation of essential proteins and lipids in the airway epithelium and sputum and the decrease in the levels of antioxidants, such as glutathione and superoxide dismutase (Drost et al., 2005; Holguin, 2013; Kirkham and Barnes, 2013).

### COPD and autophagy

The pathogenesis of COPD has been associated with an excessive increase in autophagy and mitophagy, which lead to programmed cell death of epithelial cells and emphysema (Chen et al., 2010; Gegg et al., 2010; Mizumura et al., 2014). Mutations in the ATG16L1 autophagic gene constitute a major risk factor for susceptibility to COPD. Autophagy is also increased in the lung epithelium of patients with mutations in emphysema genes, such as 1-antitrypsin deficiency (1-AT); however, its etiology is independent of smoke or particulate inhalation (Chen et al., 2008). CS has been shown to cause abnormal autophagy and mitophagy through apoptosis, leading to programmed cell death in bronchial cells (Chen et al., 2010; Mizumura et al., 2014). Studies have shown a significant increase in the levels of autophagic proteins in the lung tissues of patients with COPD at different stages of disease (Chen et al., 2008). In contrast, inhibiting autophagy by silencing LC3B protected epithelial cells from CSE-induced apoptosis (Chen et al., 2010). Moreover, the activity of histone deacetylase (HDAC) was reduced in the lungs of patients with COPD, whereas the expression of the LC3-II autophagosome formation marker and that of other autophagy-related proteins, including ATG4b, ATG5, ATG12, and ATG7, was significantly increased and associated with the increased activation of caspase-3 (Chen et al., 2008). Electron microscopy analysis of lung tissues of patients with COPD showed that the production of autophagosomes was increased in their lungs compared with to that in the lungs of the control group. The same phenomenon was also observed in animal experiments. C57BL/6 mice exposed to environmental CS for 12 weeks had increased expression of autophagic protein in their lungs (Chen et al., 2008). The lungs of CS-exposed mice exhibited increased formation of autophagosomes under electron microscopy observations, as well as increased expression of LC3B-II (Chen et al., 2008). The initial suggestion of the importance of autophagy in the progression of COPD came from studies on upstream regulators of autophagy, such as toll-like receptor 4 (TLR4) and early growth response-1 (EGR-1). In these studies, inhibition of autophagy limited *in vivo* inflammation, cell dysfunction, and apoptosis observed with chronic exposure to CS (Chen et al., 2008; An et al., 2012). Overexpression of exogenous superoxide dismutase (SOD) reduced the levels of expression of early growth response 1 (Egr-1) gene and protein, a transcription factor essential for hypoxia-related autophagy in the lungs (Nozik-Grayck et al., 2008). Studies have shown that CSE reduces the activity of HDAC in lung epithelial cells, thereby increasing the binding of Egr-1 and the E2F transcription factor to the LC3B promoter, thus increasing the expression of LC3B (Chen et al., 2008). Consequently, the CS-mediated reduction in the activity of HDAC leads to the transcriptional activation of Egr-1 and E2F-4, thereby inducing autophagic death (Chen et al., 2008). These results suggested that regulating the autophagic pathway might be beneficial in COPD interventions (Yao and Rahman, 2011). Knockdown of LC3b was reported to inhibit the activation and apoptosis of caspase-3 and improve cell viability in bronchial epithelial cells exposed to CSE, which was consistent with the view that autophagy is harmful (Chen et al., 2008). Furthermore, the increased expression of the early growth response protein 1 (EGR-1) transcription factor was shown to be necessary for increasing the levels of LC3B and ATG4B Egr-1-deficient mice exhibited a decrease in the levels of LC3B-II and ATG4B after exposure to CS, thereby mitigating the development of emphysema (Chen et al., 2008). Studies in mice exposed to CS for 16 weeks showed that CS induced autophagy in neutrophils through the activation of platelet-activating factor receptor (PAFR). Conversely, blockade of PAFR with rupatadine reduced the autophagic death of neutrophils, thereby reducing emphysema (Lv et al., 2017).

The activity of LC3B is regulated by a variety of membrane-associated and cytoplasmic factors. Studies have found that LC3B is bound to the Fas complex, a component of the DISC, in a manner dependent on the caveolin-1 caveolae-scaffolding protein. Exposure to CS has been shown to result in the rapid dissociation of LC3B from the Fas complex, consistent with the activation of the extrinsic apoptotic pathway (Nakahira et al., 2014). Accordingly, mutations in the caveolin-1 binding motif of LC3B have been reported to attenuate the proapoptotic effect resulting from the expression of LC3 (Chen et al., 2010). Deletion of the LC3B autophagic protein inhibited CS-induced airspace enlargement *in vivo* (Chen et al., 2010), while knocking down caveolin-1 (Cav-1) sensitized the epithelial cells to CS-induced apoptosis, with *Cav-1* knockout mice exhibiting higher levels of CS-induced apoptosis *in vivo* (Chen et al., 2010).

Our previous studies showed the protective effects of IP_3_R against damage in extracted smoke solution (ESS)-treated HBE cells, which was achieved by reducing oxidative stress. This result might have been related to variations in the IP_3_R-induced release of Ca^2+^, which influences the generation of mitochondrial ROS (Zhang et al., 2021). Some researchers have found that IP_3_ binds to IP_3_R on the endoplasmic reticulum and induces the release of stored intracellular Ca^2+^. High levels of intracellular Ca^2+^ activate calmodulin, thereby blocking autophagy (Harris and Rubinsztein. 2011). Some researchers have observed that CS can induce the deposition of proteases, thereby driving the accumulation of ubiquitinated proteins (aggregates) in epithelial cells and exacerbating chronic inflammation (van Rijt et al., 2012). Oxidative stress-induced increases in histone deacetylase-6 have been associated with autophagic degradation and shortening of bronchial cilia, suggesting mucociliary dysfunction (Harris and Rubinsztein. 2011). Fujii *et al.* found that CS activated autophagy in human bronchial epithelial cells isolated from patients with COPD, leading to increased cell senescence and accumulation of the p62 autophagic adaptor protein and several ubiquitinated proteins (Fujii et al., 2012). Inhibition of autophagy was shown to further increase the levels of p62 and ubiquitinated proteins (Fujii et al., 2012). Researchers have speculated that the accumulation of p62 and ubiquitinated proteins observed in the lung tissues of patients with severe COPD-emphysema suggests an insufficient autophagic clearance is involved in the pathogenesis of COPD (Tran et al., 2015).

**FIGURE 3:**
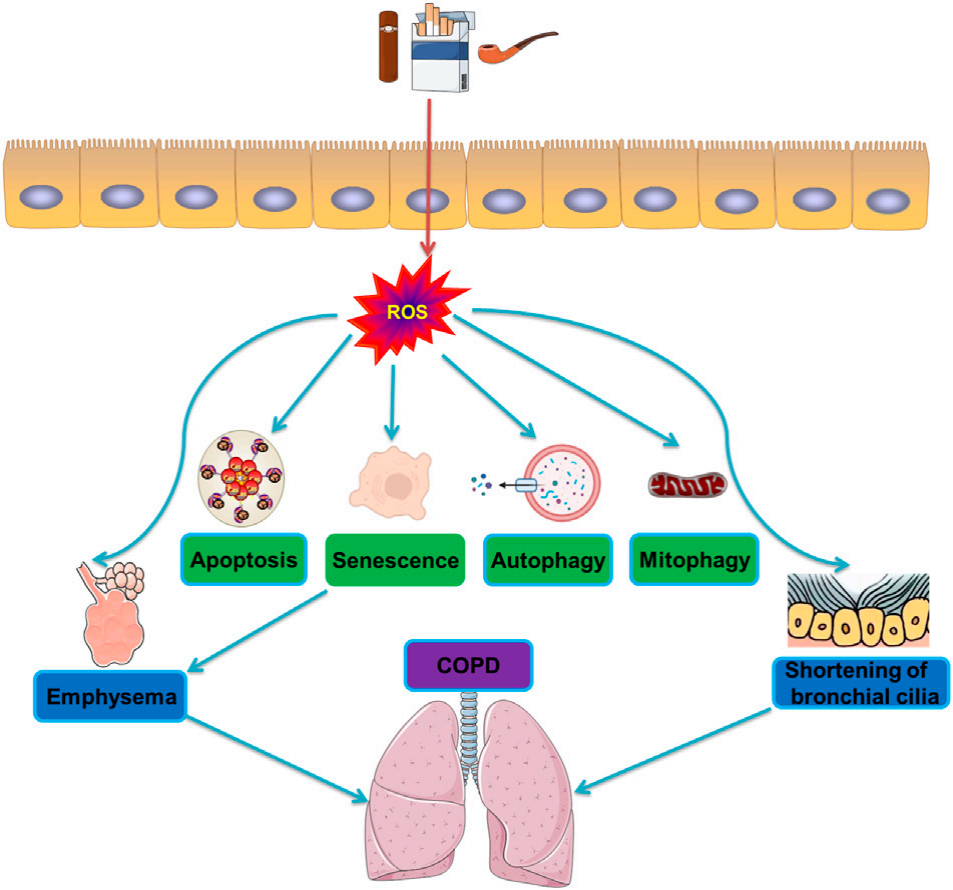
Molecular mechanisms of autophagy during oxidative stress in COPD. CS induces activation of oxidative stress and production of reactive oxygen species ROS. The generation of ROS then triggers the formation of apoptosis and senescence, and degradation of autophagy and mitophagy, leading to emphysema and shortening of bronchial cilia, and ultimately induces COPD.

**FIGURE 4:**
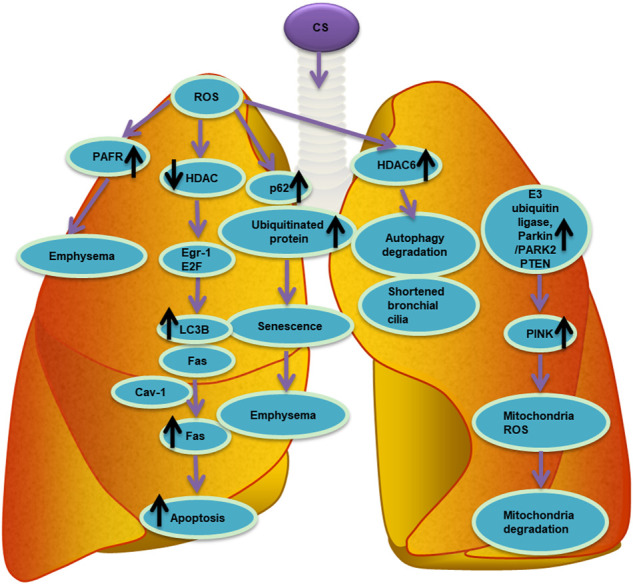
List of molecular entities involved in autophagy during oxidative stress in COPD. ROS induced by CS involve different molecular entities in apoptosis, senescence, autophagy and mitophagy in the pathogenesis of chronic obstructive pulmonary disease COPD.

Racanelli *et al.* found that the CS-induced excessive autophagy and mitophagy led to bronchial cell apoptosis and necroptosis, respectively, thereby providing a possible mechanism for the development of emphysema (Racanelli et al., 2020). The activation of mitochondrial selective autophagy, namely the connection between mitophagy and other regulated forms of cell death, such as apoptosis and necroptosis, is a driving factor of the COPD phenotype and underscores its importance in normal lung homeostasis and pathogenesis (Hou et al., 2014; Wang et al., 2017; Han et al., 2018; Wen et al., 2019; Zhang et al., 2019). CS-induced mitochondrial dysfunction and loss of mitochondrial phagocytosis have also been reported to induce cellular senescence and progression of COPD. Recent studies have shown that oxidative stress can accelerate aging by depleting stem cells, thereby causing accumulation of dysfunctional mitochondria, and decreasing autophagy, all of which generate additional oxidative stress (Mercado et al., 2015). Numerous studies have shown that CSE causes accumulation of damaged mitochondria with severe mitochondrial damage through mitophagy (Mizumura et al., 2014; Ahmad et al., 2015; Ito et al., 2015; Araya et al., 2019). Likewise, CS-induced endogenous ROS are known to stimulate the production of mitochondrial fragments in primary human bronchial epithelial cells. These mitochondrial fragments produce additional ROS, accelerating cellular aging (Hoffmann et al., 2013; Kubli et al., 2013). Induction of mitochondrial autophagy reduces the production of ROS by removing damaged mitochondria, conferring a significant protective effect on human bronchial epithelial cells (Ito et al., 2015). As such, mitochondrial phagocytosis might downregulate excessive inflammation and serve as a protective mechanism in patients with COPD (Yao et al., 2021). Studies have shown that E3 ubiquitin ligase, parkin/PARK2, and PINK1 are the primary regulators of the clearance of dysfunctional mitochondria (Dagda et al., 2009; Gottlieb and Gustafsson, 2011). Inhibiting the activation of mitochondrial phagocytosis by inhibiting either the PINK or PRKN signaling pathways led to the increased production of ROS and activation of inflammasome in small airway epithelial cells of patients with COPD (Ito et al., 2015). In general, CSE has been confirmed to induce mitochondrial dysfunction, damage mitochondrial phagocytosis, and cause accumulation of damaged mitochondrial DNA (Ahmad et al., 2015). Mitochondrial phagocytosis controls the excessive mitochondrial degradation of ROS in damaged mitochondria, and PINK1^−/−^ mice were found to be protected from CS-induced mitochondrial dysfunction (Mizumura et al., 2014). Parkin is a key regulator of mitochondrial phagocytosis and has been shown to be downregulated in tissues of patients with COPD (Ito et al., 2015). In a mouse model of COPD, loss of the parkin gene (Prkn^−/−^) resulted in exacerbation of airway thickening and airspace enlargement (Ito et al., 2015). Therefore, reversing dysfunctional mitochondrial phagocytosis should be the first choice for the treatment of COPD. In *vitro* experiments, overexpression of Parkin in epithelial cells resulted in inhibition of mitochondrial production of ROS, whereas CS extracts caused cell senescence (Araya et al., 2019). *In vivo* and *in vitro* studies showed that the increased levels of Parkin enhance mitochondrial phagocytosis and disrupt the progression of COPD (Araya et al., 2019). Autophagy is one of the most important biological responses for regulating the levels of ROS and oxidative stress in cells (Mizushima, 2018) through the clearance and degradation of damaged mitotic and oxidized proteins (Yao and Rahman, 2011). For example, sirtuin 1 (SIRT1), a type III histone deacetylase, was reported to positively regulate mitochondrial phagocytosis by upregulating the expression of the peroxisome proliferator-activated receptor-γ coactivator 1α (PGC-1α); the expression of PGC-1α decreased in the lungs of patients with moderate and severe COPD (El-Khamisy et al., 2005). Other studies have shown that the expression of PINK1, the receptor-interacting serine/threonine kinase 3 (RIPK3/RIP3) necroptosis regulator, and the DNM1L mitochondrial fission regulator increased in the lung epithelial cells of patients with COPD, indicating the widespread activation of mitochondrial phagocytosis and necroptosis under continuous CSE stimulation (Mizumura et al., 2014). This relationship between mitochondrial phagocytosis and necroptosis is essential in the progression and outcomes of patients with COPD. The molecular mechanism and molecular entities involved in autophagy during oxidative stress in COPD are summarized in Figure 3 and Figure 4 **,** respectively.

An increasing body of evidence has indicated that autophagy in a variety of cell types plays a major role in the pathogenesis of COPD. Both excessive and insufficient autophagy drives the inflammation, cell death, and cell dysfunction that are observed in COPD. Treatment options for COPD remain rather limited, and hence the potential of targeted autophagy as a treatment for COPD warrants further investigation (Racanelli et al., 2020). Currently, only a few modulators of autophagy have been evaluated for clinical use, including rapamycin (an activator of autophagy) and chloroquine or hydroxychloroquine (inhibitors of autophagy). Likewise, strategies for the treatment of COPD might involve drugs that target autophagic proteins or modulate the selective clearance and turnover of autophagy (Nakahira et al., 2014). The use of the rapamycin mTOR inhibitor showed that although increasing autophagy (to reducealveolar inflammation) after exposure to CS might be beneficial, rapamycin increased autophagy and the number of apoptotic and inflammatory cells in control mice after indoor air exposure. These findings underscored the complexity of targeted autophagy as a treatment modality for COPD (Yoshida et al., 2010). To date, the number of clinical trials evaluating autophagy modulation therapy in patients with COPD remains insufficient. This might be due to the consideration that increased autophagy is always beneficial for patients, and to the lack of a reliable method to study the effects of autophagy in patients. Another reason might be that autophagy is a controversial process, as insufficient autophagy results in aging, whereas excessive autophagy results in cell death. The discovery of therapeutic drugs that modulate autophagy in COPD is still in the early stages, and many clinically effective drugs are being repositioned to promote autophagy in COPD in *vivo* and *in vitro* models (Tan et al., 2019). However, further research is needed to critically evaluate the role of these drugs in the treatment of abnormal autophagy in COPD.

### Pre-clinical and clinical studies of antioxidant activities and regulation of autophagy in COPD

A number of natural and synthetic compounds have been shown to counteract CS-induced oxidative stress. These compounds have antioxidant activity and can be used to ameliorate chronic inflammatory and injurious responses in COPD. For example, 3, 4, 5-trihydroxyhexanostyrene (resveratrol), a plant polyphenol, has been shown to have both anti-inflammatory and antioxidant functions, inhibit CS-induced autophagy and improve the prognosis of COPD (Hwang et al., 2010; Knobloch et al., 2010; Wood et al., 2010). Spermidine is a natural polyamine that restores autophagy activity and reduces oxidative stress (de Cabo et al., 2014; Ghisalberti et al., 2016). Rapamycin can reverse defective antioxidant responses or inhibit the mTOR pathway to reduce oxidative stress damage, which can prevent aging and chronic inflammation (Mercado et al., 2015). N-acetylcysteine has anti-inflammatory and antioxidant effects (Vanella et al., 2017). These antioxidants bring new directions for the treatment of chronic inflammatory lung diseases such as COPD.

## Conclusion and perspectives

Autophagy is a generalized response to oxidative stress, intended to remove damaged subcellular substrates and maintain mitochondrial homeostasis. Autophagy serves a protective or adaptive function in the pathogenesis of disease, including metabolic or mitochondrial dysfunction, protein aggregation, inflammation, and oxidative stress. Consistently, it plays an essential role in maintaining the metabolic homeostasis of lung tissues in chronic respiratory diseases. Therefore, regulation of autophagy under oxidative stress is critical to cell homeostasis and survival. Recent studies have also shown that autophagy profoundly affects inflammation and the immune system, impacting pathogen clearance, cytokine regulation, and antigen presentation. Hence, autophagy plays a key role in human diseases associated with pro-oxidative or pro-inflammatory states. Oxidative stress is a complex phenomenon involved in the physiology and pathophysiology of many lung diseases, and a number of studies have shown that the interaction between ROS and autophagy is closely associated with the development of many lung diseases, including COPD. In this review, we presented an overview of the interplay between autophagy and oxidative stress and focused on the regulation of autophagy under oxidative stress in patients with COPD. Oxidative stress due to CS and environmental pollution plays an essential role in lung inflammation by upregulating redox-sensitive transcription factors, the induction of autophagy as well as unfolded protein response. Interestingly, ROS-induced autophagy can be both a cytoprotective mechanism alleviating oxidative stress and a destructive process. The regulation of autophagy under oxidative stress plays an essential and complex role in the pathogenesis of COPD, but the signaling pathways involved and their molecular effects remain to be defined. Studies have shown that oxidizing agents, hypoxia, and proinflammatory drugs that cause lung damage can activate autophagy, but there have been few studies on lung cells or human lung diseases to date. Appropriate modulation of autophagy is crucial for the development of new treatment strategies for COPD involving oxidative stress. Future studies might include drug screening for molecules that inhibit or induce autophagy, as well as developing autophagy inhibitors or activators and antioxidants (*e.g.*, Nrf2 activators), which might provide more avenues for the treatment of COPD.

## Data Availability

The original contributions presented in the study are included in the article/supplementary material, further inquiries can be directed to the corresponding authors.
